# The Impact of Physical Activity on Adolescent Idiopathic Scoliosis

**DOI:** 10.3390/life13051180

**Published:** 2023-05-13

**Authors:** Josipa Glavaš, Mirjana Rumboldt, Željka Karin, Roberta Matković, Vesna Bilić-Kirin, Vesna Buljan, Tanja Obelić-Babok, Jure Aljinović

**Affiliations:** 1 Department of School and University Medicine, Teaching Institute for Public Health, Split-Dalmatia County, 21000 Split, Croatia; ravnateljica@nzjz-split.hr; 2 Department of Family Medicine, University of Split School of Medicine, 21000 Split, Croatia; mirjana.rumboldt@mefst.hr; 3 Department of Pediatrics, University of Split School of Medicine, 21000 Split, Croatia; 4 Department of Mental Health, Teaching Institute for Public Health, Split-Dalmatia County, 21000 Split, Croatia; roberta.matkovic@nzjz-split.hr; 5 Department of School Medicine, Teaching Institute for Public Health, Osijek-Baranja County, 31000 Osijek, Croatia; vesna.bilic.kirin@gmail.com (V.B.-K.); vbuljan61@gmail.com (V.B.); 6 Faculty of Medicine Osijek, Josip Juraj Strossmayer University of Osijek, 31000 Osijek, Croatia; 7 Department of Preventive School Medicine, Institute for Public Health, Varaždin County, 42000 Varaždin, Croatia; 8 Institute of Physical Medicine and Rehabilitation with Rheumatology, University Hospital Split, 21000 Split, Croatia; jure.aljinovic@mefst.hr; 9 University Department of Health Studies of the University of Split, 21000 Split, Croatia

**Keywords:** scoliosis screening, school doctors, sports, forward bend test, schoolchildren

## Abstract

The prevalence of adolescent idiopathic scoliosis (AIS) is increasing, partly due to a lack of physical activity. In a cross-sectional study with 18,216 pupils (5th, 6th, and 8th grades) from four Croatian counties using the forward bend test (FBT; presumed AIS), the prevalence of AIS and its correlation with physical activity were evaluated. Pupils with presumed AIS were less physically active than their peers without scoliosis (*p* < 0.001). Abnormal FBT was more prevalent among girls than boys (8.3% vs. 3.2%). Boys were more physically active than girls (*p* < 0.001). Pupils with presumed AIS were less physically active than their peers without scoliosis (*p* < 0.001). A higher prevalence of presumed AIS was found among inactive or just recreationally active schoolchildren than among those engaged in organized sports (*p* = 0.001), girls especially. Pupils with presumed AIS were less active and had fewer weekly sports sessions than their peers without scoliosis (*p* < 0.001). Notably low prevalence of AIS was detected among pupils engaged in soccer (2.8%, *p* < 0.001), handball (3.4%, *p* = 0.002), and martial arts (3.9%, *p* = 0.006), while it was higher than expected in swimming (8.6%, *p* = 0.012), dancing (7.7%, *p* = 0.024), and volleyball (8.2%, *p* = 0.001) participants. No difference was detected for other sports. A positive correlation was found between time spent using handheld electronic devices and the prevalence of scoliosis (r_s_ = 0.06, *p* < 0.01). This study confirms the increasing prevalence of AIS, particularly among less athletic girls. Further, prospective studies in this field are required to explain whether the higher prevalence of AIS in these sports is due to referral or other aspects.

## 1. Introduction

Modern life advantages have influenced generations of children and adolescents in the past two decades by slowly substituting active recreation with screen-based ones [1,2]. This decline in physical activity among adolescents was described as a global trend [3]. 

According to the World Health Organization (WHO) Guidelines on “Physical Activity and Sedentary Behavior”, children and adolescents should have at least 60 min of moderate-to-vigorous intensity physical activity per day [4]. Additionally, exercises that strengthen muscles and bones should also be incorporated [4]. Physical inactivity is defined as not meeting these recommendations [4]. 

Adolescent idiopathic scoliosis (AIS) is considered multifactorial, and lately, there is a growing body of evidence for a lack of physical activity as one of the contributing factors to its development in schoolchildren [5,6,7]. A recent review by Tang et al. suggested that AIS development could start with soft tissue anomalies that may be modified by exercise and stretching [8]. Overall, sport is considered beneficial for AIS patients [9]. Negrini et al. suggested that regular sports activity can reduce scoliosis even in patients with braces [10]. However, certain types of sports are seemingly associated with a higher prevalence of AIS: ballet [9,11], rhythmic gymnastics [6,9,11], dance [6], volleyball [11], tennis [11], badminton [11], and swimming [9,11]. This link may be due to the referral of children with abnormal postures to some of these sports (e.g., swimming) or AIS potentiation, favoring one side of the body (e.g., tennis).

Similar to the worldwide results, the latest Croatian survey on Health Behaviour in School-aged Children in 2017/2018 (HBSC) reports the prevalence of insufficient physical activity in more than 70% of adolescents aged 11–15 and a decline in physical activity since 2002 [12]. Physical inactivity was observed more often in girls than in boys [12]. Accordingly, the latest research on the prevalence of AIS in Croatia showed an increase in the analyzed 10-year period from 4.9% to 5.8%, and its prevalence rocketed in girls [13].

Based on past research, we know that gender and physical activity level are related to AIS, and we expect that girls and physically inactive children have a higher prevalence of AIS. There has been little research into the relationship between AIS and physical activity. It is unclear whether some sports, due to their nature (e.g., asymmetrical or symmetrical), present a higher risk for AIS development, whether there are gender differences in AIS occurrence in certain sports, and whether lower or higher physical activity levels influence its development. This investigation was designed to assess the prevalence of presumed scoliosis (positive forward bend test) regarding the level of physical activity, and to evaluate the impact of different sports on the occurrence of presumed AIS. 

## 2. Materials and Methods

### 2.1. Participants and Study Design

This cross-sectional study included 18,216 elementary school pupils (5th, 6th, and 8th grades), aged around 11, 12, and 14 years, respectively, from four counties in Croatia. FBT, a simple and reliable screening test for AIS with a positive predictive value of 84.7% [13], is standard in our country. Children with a positive test determined by the school medicine specialists during routine systematic examinations (*n* = 1053) were considered cases (presumed scoliosis). Controls were children with a negative FBT (*n* = 17,163). The Ethical Committee of the Institute of Public Health approved this research (Class: 500-01/19-01/7; Registry number: 2181-103-01-19-1). Informed consent and assent were obtained from parents and children, respectively.

### 2.2. Procedures

The obtained data were noted in survey forms designed for this study, comprising information related to medical records (age, gender, class, family history of scoliosis, physical activity, sedentary behavior), anthropometric data, clinical signs indicating scoliosis (particularly FBT), and respective measures undertaken by specialists. As for the physical activity level, it was recorded whether the pupils were engaged in organized sports [14], irregularly (recreationally) active, or physically inactive. Additional information on the duration and frequency of sports activity was also noted. Recreational activities were classified as irregular physical activities since they do not meet the criteria for frequency, duration, and intensity of training as in sports activities supervised by an adult. The data were collected during the school year 2019–2020.

### 2.3. Statistical Analysis

All statistical analyses were performed using the IBM SPSS 20.0 package (IBM Corp., Armonk, NY, USA). Descriptive statistics for proportions are presented as percentages, mean, and standard deviation. The following nonparametric tests were used to evaluate differences/relationships between the observed variables: the χ^2^ test, the Mann-Whitney U test, and Spearman’s rank correlation, as appropriate. *p*-values < 0.05 were considered statistically significant.

## 3. Results

A total of 18,216 pupils participated in the study, 50.1% male and 49.9% female (Table 1). A positive FBT was found in 1053 (5.8%) of them. These examinees were notably slimmer and taller than their nonscoliotic counterparts. Concerning physical activity, there were 4277 (23.5%) inactive children, 4912 (27.0%) recreationally active children, and 9027 (49.5%) engaged in organized sports (Table 2). 

A gender difference was noted regarding the level of physical activity (Table 2). Boys were physically more active than girls, and this difference was statistically significant (Mann-Whitney U test, *p* < 0.001). 

A higher prevalence of presumed scoliosis was observed among inactive or recreationally active girls than among those in organized sports (Table 2), and this difference was statistically significant (Mann-Whitney U test, *p* = 0.001). The difference in presumed AIS prevalence between boys according to their physical activity level was not statistically significant (Mann-Whitney U test, *p* = 0.056). Still, in the subgroup of inactive boys, the prevalence of presumed AIS was significantly higher than among those engaged in organized sports (χ^2^ = 5.311, df 1, *p* = 0.021). A higher prevalence of presumed AIS was also observed in the subgroup of inactive girls compared to girls engaged in organized sports (χ^2^ = 12.954, df 1, *p* < 0.001).

Combined, without regard to gender, pupils with presumed AIS were less active than those who had no scoliosis (Mann-Whitney U test, *p* < 0.001). Furthermore, pupils with presumed scoliosis had fewer weekly sports sessions than those without scoliosis (Mann-Whitney U test, *p* < 0.001). There were 63.4% (*n* = 279) of pupils with scoliosis engaged in organized sports for less than 3 h weekly and 36.6% (*n* = 161) of them with 3 h or more of weekly sports activities. Among those without scoliosis, 61.1% (*n* = 5245) practiced sports for less than 3 h weekly, while 38.9% (*n* = 3334) had more than 3 h of weekly sports sessions. This analysis included 18,208 pupils (8 had missing information on the type of sports activity and its frequency), 49.5% (*n* = 9019) of pupils involved in organized sports, and 50.4% (*n* = 9189) of those not involved in some kind of formal sports practice. 

Our participants were engaged in 31 sportive activities, but only 11 sports were reported by more than 1% of the examinees and included in further analysis (Table 3). Dancing (χ^2^ = 5.092, df 1; *p* = 0.024), swimming (χ^2^ = 6.376, df 1; *p* = 0.012), and volleyball (χ^2^ = 11.144, df 1; *p* = 0.001) had a significantly elevated prevalence of presumed AIS. A lower prevalence of a presumed AIS was observed for martial arts (χ^2^ = 7.670, df 1; *p* = 0.006), soccer (χ^2^ = 54.604, df 1; *p* < 0.001), and handball (χ^2^ = 9.467, df 1; *p* = 0.002). The prevalence of AIS in other sports was marginal: tennis (χ^2^ = 0.145, df 1; *p* = 0.703), gymnastics (χ^2^ = 0.736, df 1; *p* = 0.391), basketball (χ^2^ = 1.008, df 1; *p* = 0.315), athletics (χ^2^ = 0.791, df 1; *p* = 0.374), and water polo (χ^2^ = 0.065, df 1; *p* = 0.798).

Correlations between various sports and gender (Table 4) indicate that girls predominantly practice gymnastics (r_s_ = 0.114, *p* < 0.01), volleyball (r_s_ = 0.220, *p* < 0.01), and dancing (r_s_ = 0.182, *p* < 0.01), while more boys than girls practice soccer (r_s_ = −0.355, *p* < 0.01).

Sedentary screen time, such as watching TV, playing computer games, and using mobile phones for internet browsing, was prominent. The average screen time for all devices with a screen (overall screen time) was 3 h per day (M = 3.01, SD = 1.90). Some children perceived that they spent up to 15 h using devices with screens (*n* = 2), while some did not seem to use these devices at all (*n* = 1550; i.e., 8.5% of the sample). Overall, 38.4% of our pupils do not watch TV, and 42.8% do not play computer games. The data on time spent on handheld devices was available for only 37.4% of the participants (*n* = 6813). Correlations between presumed AIS and recreational screen time (Table 5) indicate that mobile phones were mostly used (r_s_ = 0.62, *p* < 0.01). More time spent with handheld devices was associated with a higher prevalence of scoliosis; this correlation was significant but weak (r_s_ = 0.06, *p* < 0.01). 

## 4. Discussion

This study investigated the associations between physical activity and presumed AIS. As expected, we found that physically inactive adolescents, particularly girls, are more prone to AIS. The affected examinees were significantly taller and thinner than their nonscoliotic counterparts. In addition, we observed a higher prevalence of presumed AIS in children who participated in fewer weekly sports sessions. There was no link between asymmetrical sports and AIS. 

Croatian schools have compulsory physical education three times per week in grades 1–3, twice per week for higher grades of primary school, and 1–2 times per week in high school. Still, insufficient activity levels among our adolescents prevail. Schools are regarded as ideal settings for promoting physical activity among children due to their broad reach because school-aged children spend a substantial amount of their time in the school setting. However, a recent review found that school-based physical activity interventions, as currently designed and delivered, probably have little to no impact on overall time spent in moderate to vigorous physical activity and may have little to no impact on time spent sedentary [15]. It is estimated that not getting enough exercise (physical inactivity) causes up to 5.3 million deaths globally, and this is a major risk factor for the majority of chronic diseases and cancers [15]. This is concerning, especially given that physical exercise patterns in childhood can lead to similar patterns in adulthood [15]. There is much to grasp regarding the possible risk factors associated with AIS, but physical activity seems to be one of those modifiable risk factors. As suggested by Neil-Sztramko et al., an additional focus on physical activity outside the school environment may help to increase overall physical activity levels [15]. Therefore, our preventive strategies encourage physical activity. Another preventive tool in our country is continuous scoliosis surveillance, covering the main growth stages [9,13]. Even though FBT is not perfectly accurate, it proved its worth in screening large samples of schoolchildren [13].

Our results show that half of our participants were inactive or irregularly active. Boys were more physically active than girls, but there was no marked difference in the prevalence of AIS between active and less active boys, while in girls, it was much higher in less active girls. This physical inactivity trend is recognized at a global level [3] and at a national level too [12]. Croatia has been performing the HBSC survey since 2002, and these gender and activity differences are constant. Following the decline in physical activity throughout the years [3,12], an increase in AIS prevalence over 10 years was noted, especially in girls [13]. Our findings, along with HBSC’s results among Croatian schoolchildren, should entice more engagement in physical activity promotion by our school doctors, particularly because growing numbers of children seek absolution from physical education in our schools, for which school doctors give permission. Moreover, girls with scoliosis are paradoxically often exempted from physical education lessons due to their spinal deformities [16]. Therefore, promotional measures should target girls in particular because they tend to participate less than boys in vigorous physical activities. To be equally active as boys, the same level of opportunity through accessible activities should be provided for them in local communities. 

Our data reconfirm the notion that children with scoliosis are less active than their peers [5,7], despite the fact that Diarbakerli et al. found that adolescents with scoliosis and adolescents without AIS have comparable levels of physical activity [17]. It could be due to fear of injury or that sports might negatively influence scoliosis progression. Furthermore, children with AIS may feel less confident conducting physical activities and may not participate as much as other children, according to McMaster et al. [18]. Still, most studies support physical activity for patients with scoliosis [19]. Although the SOSORT 2016 guidelines suggest that sport has no major favorable impact on AIS progression, it is recommended for overall well-being and fitness level [9]. Furthermore, recent findings show that regular sports activity can even reduce the Cobb angle in children with braces [10]. Even children after spinal arthrodesis can safely return to any sport [9,19,20,21]. Since there is no evidence that physical or sporting activity is harmful to patients with scoliosis [19], the role of school doctors and other physicians is to reassure their patients that they should resume an active lifestyle and that scoliosis is not a reason to avoid exercise. 

The number of weekly sports sessions is just as important as engaging in sports per se. Our AIS examinees were not only less engaged in organized sports, but they also had fewer weekly sporting activities of any kind. A higher prevalence of AIS among pupils practicing physical activity for less than 3 h weekly was already noted by Scaturro et al. [6]. The necessary amount of such activity varies with age, as distinctly described for children and adolescents aged 5–17 by WHO 2020 guidelines [4]. In children and adolescents, there is a dose-response relationship between physical activity, sedentary behavior, and health-related quality of life. A recent study provided evidence that more time spent engaging in higher-intensity physical activities is associated with better health outcomes, even though the optimal physical activity dose cannot be determined precisely [22]. Indeed, a prospective study has shown that children performing four or more episodes of moderate/vigorous physical activity per week were less likely to develop scoliosis [7]. Negrini et al. found that full-time braced adolescents showed an increase in the odds of improvement with the rise of sports frequency [10]. 

In 2020, the WHO included for the first time recommendations on sedentary time in its guidelines. The greater the time spent in sedentary behavior, especially recreational screen time, the poorer the health outcomes in children and adolescents [4]. This association is generally stronger for television viewing or recreational screen time [4,22]. Thus far, limited evidence suggests that sedentary behavior is not related to bone health in children and adolescents [4,22]. Although our scoliotic pupils spent significantly more time on handheld devices than the controls, the correlation with presumed scoliosis was unexpectedly weak. There is insufficient evidence on time limitations for screen time, but the guidelines recommend limiting sedentary recreational screen time to no more than 2 h per day [4,22]. Nevertheless, our participants spent on average 3 h per day using such devices, and the perceived screen time for some of them reached even 15 h. To change screen-time habits, alternative activities should take place. Lissak proposes large-scale programs in communities to fill free time since the daily obligations of parents often make it difficult to provide children with non-screen-based alternatives [23]. Physical activity is one way to spend one’s free time. 

We have observed that some sports are associated with a higher prevalence of AIS. The answer to whether the higher occurrence is a matter of referral to these sports or whether certain sports have an impact on scoliosis development remains elusive. Typically, some sports are criticized for their asymmetrical nature. However, some degree of asymmetry is present in all sports, even those considered mostly symmetrical, and each person typically has a dominant side [24]. Our results support the published findings of elevated scoliosis frequency in swimming [9,11], volleyball [11], and dancing [6]. Indeed, volleyball could be considered an asymmetrical sports activity, and even dancing has unilateral loading and high-intensity unilateral movements [24]. On the contrary, swimming is one of the sports habitually recommended for children with scoliosis. It is considered a rather safe activity that benefits the whole body since it engages almost every muscle group. The observed high AIS prevalence (8.6%) among swimmers can hardly be explained by asymmetrical development facilitation. We believe it is mostly due to selection bias, i.e., referral by public opinion or professional advice. We had 321 examinees engaged in gymnastics, and, contrary to the literature [6,9,11,25], we found no significant association with AIS, despite the putatively long-lasting, asymmetrical loading of the spine [25,26]. Similar to our results, Meyer et al. found that scoliosis in gymnasts is associated with higher joint laxity and is not generated by exercise [27]. A high prevalence of scoliosis among our dancers is consistent with the published results [6,11,28], but findings from a longitudinal study indicate that dancing is negatively associated with AIS [18]. Similar to gymnasts, the pupils may have chosen dance as their bodies seem to be better suited for such activity [28], or they may have been referred to these sports.

Tennis is one of the sports charged with asymmetry as it preferentially stimulates the dominant side muscles [9,11,24]. Thus far, in our study, it has not been associated with scoliosis. The most prevalent sport in our investigation was soccer, with 15.2% of active participants. Although soccer is a hybrid sport [24], with both cyclic and acyclic movements and noted lateral asymmetries [24], we have identified less AIS than in other sports. Similar results were noted for another hybrid sport, handball. As for martial arts, although there are differences in the degree of asymmetry in various disciplines [29], the occurrence of a presumed AIS was remarkably low. The martial arts potentially favor one side of the body over the other [29], but the coach’s role is to correct the disbalance and increase skill and strength on both sides of the body. 

There were a few limitations to our study, offering ideas for future research. First, an observational study is not appropriate for the evaluation of the causative relationship between AIS and sports; it just assessed the prevalence of presumed AIS in different sports within a large study group. The AIS diagnosis was based on the Adams test alone, and in the absence of confirmatory radiographic imaging of scoliosis, the actual contribution of physical activity to AIS development may be overestimated. Second, we did not investigate the nature of enrollment in particular sports, e.g., the parents’, teachers’, or physicians’ referral, so this could be the next step for upcoming research. Furthermore, instead of a questionnaire, a more reliable measure of screen time (validated digital-screen exposure questionnaires or smartphone screen time applications) should be used. Finally, the surprisingly low correlation between leisure time spent with electronic gadgets and AIS may be due to the cross-sectional nature of our study, to prejudiced questionnaire answers (reporting bias), to a low response rate (abstinence or nonrespondent bias: over 60% of the examinees have not answered that question), or to recall bias because of the polling circumstances.

## 5. Conclusions

This study and earlier investigations [13] reveal an increasing prevalence rate of presumed AIS, mostly among inactive or just recreationally active pupils, particularly girls. Although presumed scoliosis prevalence was higher in certain sports, we could not establish the link between the predominant character of physical activity (e.g., asymmetrical vs. symmetrical) and scoliosis. For that reason, the exclusion of certain sports seems unfair. The observed correlation between scoliosis and time spent on devices with screens was astonishingly low, compelling further, specifically designed investigations.

The practice of sports in general, whether symmetrical or asymmetrical, should be encouraged in children. An initial assessment before participation in sports and a later, continuous observation for spine deformities should be one of the preventive strategies used by school doctors to stimulate physical activity, especially in children with scoliosis, as a healthy way of spending leisure time and as an alternative to time-consuming screen gazing. 

## Figures and Tables

**Table 1:** Characteristics of the examined groups.

	No AIS		Presumed AIS		Total	
	N	(%)	N	(%)	N	(%)
Gender						
Male	8836	(96.8)	295	(3.2)	9131	(100)
Female	8327	(91.7)	758	(8.3)	9085	(100)
Total	17,163	(94.2)	1053	(5.8)	18,216	(100)
Anthropometric data	Mean ± SD		Mean ± SD		*p*-value	
Height	160.32 ± 10.78		164.23 ± 9.79		<0.001	
Weight	53.04 ± 14.35		53.13 ± 12.14		0.84	
BMI (kg/m^2^)	20.39 ± 4.09		19.54 ± 3.37		<0.001	

Abbreviations: N—number of pupils, %—percentage, AIS—adolescent idiopathic scoliosis, BMI—body mass index.

**Table 2:** Prevalence of presumed AIS according to physical activity level.

Category	Active		Recreational		Inactive		Total
	N	(%)	N	(%)	N	(%)	N
Presumed AIS							
Male	155	(3.0)	66	(3.0)	74	(4.1)	295
Female	286	(7.4)	224	(8.2)	248	(10.0)	758
No AIS							
Male	5017	(97.0)	2098	(97.0)	1721	(95.9)	8836
Female	3569	(92.6)	2524	(91.8)	2234	(90.0)	8327
Total	9027		4912		4277		18216

Abbreviations: AIS—adolescent idiopathic scoliosis, M—male, F- female, N—number of pupils.

**Table 3:** Prevalence of presumed AIS in various sports activities.

Sports	Presumed AIS		No AIS		Total	
	N (M/F)	(%)	N (M/F)	(%)	N	(%)
Volleyball	83 (1/82)	(8.2)	935 (49/886)	(91.8)	1018	(100)
Swimming	36 (13/23)	(8.6)	381 (235/146)	(91.4)	417	(100)
Dancing	55 (0/55)	(7.7)	658 (36/622)	(92.3)	713	(100)
Athletics	15 (4/11)	(7.2)	193 (62/131)	(92.8)	208	(100)
Basketball	44 (18/26)	(6.7)	615 (428/187)	(93.3)	659	(100)
Tennis	15 (4/11)	(6.4)	221 (94/127)	(93.6)	236	(100)
Water polo	11 (10/1)	(5.4)	194 (182/12)	(94.6)	205	(100)
Gymnastics	15 (1/14)	(4.7)	306 (23/283)	(95.3)	321	(100)
Martial arts	41 (15/26)	(3.9)	1022 (611/411)	(96.1)	1063	(100)
Handball	28 (14/14)	(3.4)	807 (508/299)	(96.6)	835	(100)
Soccer	77 (65/12)	(2.8)	2703 (2490/213)	(97.2)	2780	(100)
Total	420 (145/275)	(5.0)	8035 (4718/3317)	(95.0)	8455	(100)

Abbreviations: AIS—adolescent idiopathic scoliosis, M—male, F—female, N—number of pupils.

**Table 4:** Correlations between presumed AIS and various sports.

	Gender	Soccer	Swimming	Tennis	Gymnastics	Martial Arts	Volleyball	Handball	Basketball	Athletics	Dancing	Water Polo	Presumed AIS
Gender	1.000												
Soccer	−0.355 **	1.000											
Swimming	−0.029 **	−0.065 **	1.000										
Tennis	0.020 **	−0.049 **	−0.018 *	1.000									
Gymnastics	0.114 **	−0.057 **	−0.021 **	−0.015 *	1.000								
Martial arts	−0.044 **	−0.106 **	−0.038 **	−0.029 **	−0.033 **	1.000							
Volleyball	0.220 **	−0.103 **	−0.037 **	−0.028 **	−0.033 **	−0.061 **	1.000						
Handball	−0.054 **	−0.093 **	−0.034 **	−0.025 **	−0.029 **	−0.055 **	−0.053 **	1.000					
Basketball	−0.068 **	−0.082 **	−0.030 **	−0.022 **	−0.026 **	−0.048 **	−0.047 **	−0.042 **	1.000				
Athletics	0.040 **	−0.046 **	−0.016 *	−0.012	−0.014	−0.027 **	−0.026 **	−0.024 **	−0.021 **	1.000			
Dancing	0.182 **	−0.086 **	−0.031 **	−0.023 **	−0.027 **	−0.050 **	−0.049 **	−0.044 **	−0.039 **	−0.022 **	1.000		
Water polo	−0.093 **	−0.045 **	−0.016 *	−0.012	−0.014	−0.027 **	−0.026 **	−0.023 **	−0.021 **	−0.011	−0.022 **	1.000	
Presumed AIS	0.110 **	−0.055 **	0.019 *	0.003	−0.006	−0.021 **	0.025 **	−0.023 **	0.007	0.007	0.017 *	−0.002	1.000

Abbreviations: AIS—adolescent idiopathic scoliosis; ** correlation significant at the 0.01 level; * correlation significant at the 0.05 level (2-tailed).

**Table 5:** Correlations between presumed AIS and recreational screen time.

	Gender	Television Viewing	Playing Computer Games	Mobile Phone/Internet Usage	Overall Screentime	Presumed AIS
Gender	1.000					
Television viewing	−0.029 **	1.000				
Playing computer games	−0.311 **	0.106 **	1.000			
Mobile phone/internet usage	0.141 **	0.023	−0.032 **	1.000		
Overall screentime	−0.139 **	0.521 **	0.579 **	0.623 **	1.000	
Presumed AIS	0.110 **	−0.011	−0.021 **	0.066 **	−0.017 *	1.000

Abbreviations: AIS—adolescent idiopathic scoliosis; * correlation significant at the 0.05 level; ** correlation significant at the 0.01 level (2-tailed).

## Data Availability

Data are available upon request.
